# Beyond Needling: Integrating a Bayesian Brain Model into Acupuncture Treatment

**DOI:** 10.3390/brainsci15020192

**Published:** 2025-02-13

**Authors:** Beomku Kang, Da-Eun Yoon, Yeonhee Ryu, In-Seon Lee, Younbyoung Chae

**Affiliations:** 1 Department of Meridian and Acupoints, College of Korean Medicine, Kyung Hee University, Seoul 02447, Republic of Korea; beomkukang@khu.ac.kr (B.K.); yoonda05@khu.ac.kr (D.-E.Y.); inseon.lee@khu.ac.kr (I.-S.L.); 2 KM Science Research Division, Korea Institute of Oriental Medicine, Daejeon 34054, Republic of Korea; yhryu@kiom.re.kr

**Keywords:** acupuncture, Bayesian brain, individual difference, predictive coding

## Abstract

Acupuncture is a medical tool in which a sterile needle is used to penetrate and stimulate a certain body area (*acupoint*), inducing a series of sensations such as numbness, dullness, or aching, often referred to as *de-qi*. But is that all? In this article, we adopt a Bayesian perspective to explore the cognitive and affective aspects of acupuncture beyond needling, specifically, how the body integrates bottom-up sensory signals with top-down predictions of acupuncture perception. We propose that the way in which we discern acupuncture treatment is the result of predictive coding, a probabilistic, inferential process of our brain. Active inference from both prior experience and expectations of acupuncture, when integrated with incoming sensory signals, creates a unique, individual internal generative model of our perception of acupuncture. A Bayesian framework and predictive coding may, therefore, aid in elucidating and quantifying the cognitive components of acupuncture and facilitate understanding of their differential interactions in determining individual expectations of treatment. Thus, a perception-based Bayesian model of acupuncture presented in this article may expand on how we perceive acupuncture treatment, from simply inserting needles into our body to one that encompasses a complex healing process supported by belief and hope of regaining health. By exploring how cognitive factors influence individual responsiveness to acupuncture treatment, this review sheds light on why acupuncture treatment is more effective in some individuals than in others.

## 1. Introduction

The term “unconscious inference” was first coined by Helmholtz in 1867 in his book about visual illusions. The development of Bayesian methods has provided solutions for explaining computational theories of perception and sensorimotor control. In computational neuroscience, this led to the Bayesian coding hypothesis, which proposes that the brain represents sensory information as probability distributions [1]. The Bayesian approach to interpreting human perception (or active inference) is consistent with the free-energy principle, which states that any self-organizing system strives to minimize its free energy [2,3]. Extrapolation of the free-energy principle yields the Bayesian brain hypothesis, in which Bayesian probability theory is used to frame perception as a constructive process based on hierarchical generative models. The hypothesis postulates that the brain is an inference machine that actively generates predictions by forming probabilistic models that create descending predictions (*prior*), against which ascending sensory signals (real-world information or *likelihood*) can be tested. This process is referred to as predictive coding. The resulting discrepancy between *prior* and *likelihood* is recognized as a prediction error, which is then forwarded to higher levels of the generative model to adjust predictions and parameters and to minimize subsequent prediction errors by updating prior belief. The brain’s motivation to minimize prediction error can be understood in the context of the free-energy principle, in which mismatch (prediction error) serves as free energy, with the brain acting to reduce prediction errors by the innate drive to minimize free energy [2].

The Bayesian framework was first applied to the visual system and has since gained acceptance across a wide range of research areas, including auditory, interoceptive, neuropsychiatric, and even social fields [4,5,6,7]. However, to our knowledge, no research or review has yet to integrate the Bayesian framework into acupuncture treatment. In this article, we provide a detailed description of the Bayesian brain model as applied to acupuncture perception. We then propose future directions of research for elucidating the neural mechanisms and contributions of cognitive factors in acupuncture perception based on predictive coding theory.

This paper adopts a conceptual and narrative approach to design a Bayesian model for acupuncture. Our methodology involves synthesizing existing evidence from neuroscience, psychology, and acupuncture research to bridge gaps in understanding how cognitive and affective processes influence acupuncture perception and efficacy. To integrate the Bayesian framework into acupuncture, we conducted a narrative review of the literature on predictive coding, cognitive appraisal, and acupuncture mechanisms, focusing on the impact of cognitive factors on acupuncture. This approach allowed us to integrate interdisciplinary perspectives into a comprehensive theoretical framework that explains how acupuncture functions within the body and brain. Our aim is to provide an overview of how the Bayesian framework can be integrated into acupuncture treatment. By examining the role of cognitive factors in individual responsiveness to acupuncture, this review highlights why acupuncture is more effective for some individuals than others.

## 2. The Components of Acupuncture from a Bayesian Brain Perspective

That there is much more to acupuncture than insertion and stimulation of the acupuncture needle has long been recognized [8]. The neural mechanisms in the brain invoked by acupuncture have been explored using brain imaging techniques [9]. Subsequent studies have identified the involvement of various cognitive factors in acupuncture treatment, such as the context of treatment [10], affective touch [11], the doctor–patient relationship [12,13], treatment expectations [14,15], and bodily attention [16].

A good example of how cognitive (non-specific) factors affect acupuncture perception is offered by the concept of the rubber hand illusion. Acupuncture stimulation delivered to a rubber hand—which participants experience as their own by enhancing bodily ownership—was shown to result in brain activation as well as a *de-qi* sensation [17]. Similarly, when acupuncture was applied to an embodied prosthetic hand vs. to a fake hand in an amputee patient, brain activation was induced only by acupuncture stimulation of the embodied prosthetic hand (which amputee patients perceive as their own); stimulation of the fake hand resulted in brain activation only in visual areas [17]. These results suggest that the cognitive aspects of acupuncture play a major role in the processing of acupuncture treatment, including an embodied rubber hand or a prosthetic hand. They further demonstrate that the perception of acupuncture can be calibrated by implicitly using a representation of one’s own body [18].

Treatment expectation also plays a crucial role in how we perceive acupuncture. For example, we may perceive acupuncture as effective because we expect it to be. Patients with chronic pain anticipating better treatment results were shown to respond more favorably to acupuncture treatment [19]. In patients experiencing knee pain, enhancing anticipation through expectancy manipulation substantially augmented acupuncture’s therapeutic effectiveness [20]. In another study [21], regardless of actual treatment allocation, patients who “believed” that they had received active acupuncture treatment reported significantly greater pain relief following third molar surgery than those who “believed” they had received placebo acupuncture treatment. This suggests that non-specific acupuncture-related factors, such as patients’ treatment perceptions and expectations, are essential to boosting the efficacy of acupuncture analgesia [21].

The contribution of treatment expectation to treatment success has been noted even outside the scope of acupuncture. In both experimental studies and clinical trials, open-label placebos, in which patients were informed that they were receiving a placebo, demonstrated remarkable efficacy [22,23,24]. Notably, both open-label placebo acupuncture and pills showed significant analgesic effects that were influenced by treatment expectations and by the identity of the practitioner [25].

Research in the fields of video-guided acupuncture imaginary treatment (VGAIT) [26], visual acupuncture [27,28], and phantom acupuncture [29] has shown that clinical efficacy is possible even without a direct tactile somatosensory afferent signal, which implies that the expectation of sensation or treatment can override actual sensory perception. Similarly, in individuals with irritable bowel syndrome, an augmented doctor–patient relationship was found to heighten expectancy significantly and, thus, also the placebo effect of sham needles [30]. How can we understand this phenomenon, wherein, because we expect a process to be efficient, it is indeed so?

Predictive coding theory addresses this question in Bayesian terms. Even without acupuncture needle insertion or stimulation, the inferred sensation of acupuncture is perceived as an effective treatment, as evidenced by the experiencing of *de-qi* in our own body, although only a rubber hand is being stimulated [17]. A plausible explanation is that our brain makes active inferences in the context of treatment, i.e., that treatment will induce *de-qi* sensations and will be efficient. In some cases, our brain assigns more importance to these predictions, thus overriding what our senses actually experience. Although the free-energy principle has been invoked to account for this phenomenon, it is unclear why our brain behaves as it does. However, from a Bayesian perspective, it can be inferred that the cognitive factors associated with acupuncture play a crucial role in how we perceive acupuncture. Thus, a perception-based Bayesian modeling of acupuncture can shed light on how and why cognitive factors influence acupuncture treatment experiences.

## 3. A Bayesian Brain Model of Acupuncture Perception

**Figure 1:**
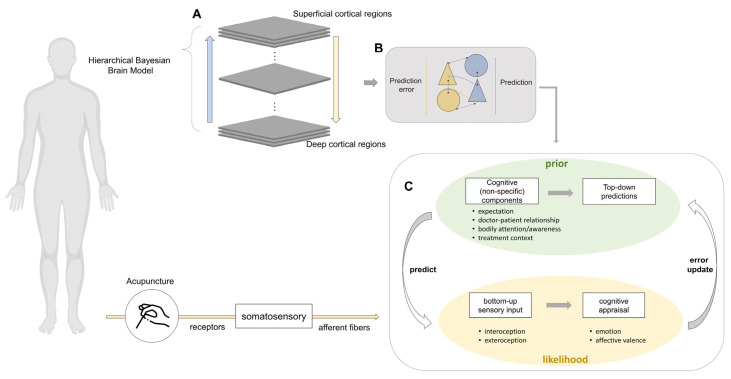
Bayesian model of acupuncture perception. (**A**) A hierarchical Bayesian internal model of acupuncture perception. In the application of predictive coding to acupuncture perception, our beliefs or expectations act as *prior*. The *prior* lays the foundation from which top-down predictions are generated. During acupuncture treatment, sensory input is perceived through receptors, sending signals to our brain via ascending neural pathways. We then assign *affective valence* to the new information, which is depicted as *cognitive appraisal* or *f* function. Finally, the cognitively appraised sensory input (likelihood) is compared with top-down predictions to form a prediction error. The latter is then weighted by gain, which defines how much influence the prediction error has on prior through factors such as attention. Bottom-up sensory input received from various receptors enters cortical regions via afferent fibers and is subjected to cognitive appraisal, constituting likelihood. Priors are then compared with likelihood to form prediction errors that are used to update internal models. (**B**) Schema of hierarchical predictive coding in a single layer. Pyramidal (prediction) neurons (triangles) interact with inhibitory neurons (circles) to project bottom-up sensory information and top-down predictions. Yellow arrows indicate prediction errors, and blue arrows the predictions. (**C**) Interplay between prior and likelihood in acupuncture perception. Prior consists of cognitive (non-specific) components of acupuncture, such as expectation, doctor–patient relationship, bodily awareness, and treatment context, and give rise to top-down predictions. Likelihood is formed by integrating bottom-up sensory input (new information) with cognitive appraisal (the process of giving a certain valence to perceived sensations). The discrepancy between prior and likelihood is referred to as the prediction error. Prediction errors are weighted by gain (learning rate), which then updates the prior (error update). Posterior: This refers to the outcome of Bayesian prediction in the perception of acupuncture. It is formed by integrating the prior with the prediction error to shape our understanding of a particular afferent stimulus. The posterior then becomes a new prior when the next stimulus is encountered. Prediction error: This is the discrepancy between the prior and the likelihood. In the context of acupuncture perception, it represents the difference between what we expect (prior) and what we actually sense (likelihood), resulting in the experience of a prediction error. Typically, prediction error is generated by comparing sensory input (which has been cognitively appraised) with the prior. The figure was modified from Seth et al. [6].

Predictive coding theory suggests that the brain is able to predict incoming sensory inputs and minimize errors through continuous updates of prior knowledge [2], in which bottom-up sensory data, top-down expectations derived from past experiences, and contextual cues are integrated to formulate predictions of somatic experiences [31,32]. Predictive coding can, therefore, aid in deciphering how the brain processes emotions, pain, and placebo analgesia [33,34,35]. Perceptions of needling sensations and therapeutic efficacy in acupuncture are similarly influenced by prior experiences and treatment expectations [27,36] (Figure 1). Hence, even in the absence of a physical stimulus, acupuncture elicits valid physiological responses, such as brain activation, as well as psychophysiological responses [16,37]. In other words, how we sense acupuncture and perceive its efficacy may vary depending on our prior experiences and our treatment expectations [38]. The individual nature of both these factors is the cornerstone of a Bayesian brain model of acupuncture perception.

The Bayesian brain model of acupuncture perception can be expressed as follows:

$${Posterior}_{acu}(t)={Prior}_{acu}(t)+\eta \;*\;PE(t)$$

$$PE(t)=|f(sensory\;input)-{Prior}_{acu}\left(t\right)|$$

$${Prior}_{acu}(t+1)={Posterior}_{acu}(t)$$

η = gain (learning rate)

*PE* = prediction error

*f* = cognitive appraisal

*t* = time (temporal)

When predictive coding is applied to acupuncture perception, our belief in the potential of acupuncture acts as the *prior* (${Prior}_{acu}(t))$. The *prior* mainly refers to what we predict the acupuncture experience to be and consists of various cognitive (non-specific) acupuncture-related factors, such as treatment expectation, medical context, and the doctor–patient relationship. These cognitive components lay the foundation for top-down predictions, i.e., the prior, are generated. During acupuncture treatment, sensory input is perceived through receptors, generating signals that are sent to our brain via ascending neural pathways. This initiates a process of cognitive appraisal (represented as the function *f*) in which affective valence is assigned to the new sensory information. The appraised sensory input ($f\left(sensory\;input\right),$ in Bayesian terms, *likelihood*) is compared with top-down predictions to form a prediction error (PE(t)). The prediction error is then weighted by the gain (η or learning rate), which defines how much influence the prediction error has on the prior. Bodily attention can be a good example of how much weight a person gives to afferent information (e.g., acupuncture stimulation) [39]. The prior is then integrated with the weighted prediction error, updating the former prior and conclusively forming the posterior (${Posterior}_{acu}(t))$. During the next treatment, the posterior naturally becomes the prior ((t+1)), and the whole sequence is repeated.

**Figure 2:**
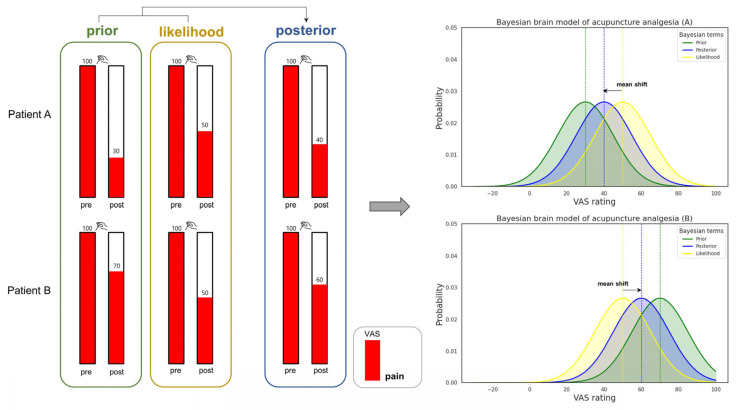
Visualization of the Bayesian brain model applied to acupuncture analgesia. We consider a hypothetical scenario where Patient A and Patient B experience acupuncture analgesia. The red box indicates perceived pain, with Visual Analogue Scale (VAS) ratings displayed numerically above. Different priors are assigned to Patient A and Patient B, as shown in the green-outlined box. This disparity may arise from various factors, such as different experimental conditioning or contrasting real-life experiences and expectations regarding acupuncture. For instance, Patient A and Patient B might expect different levels of analgesia due to prior conditioning or past experiences—Patient A may have had a positive experience with significant pain relief, while Patient B’s experience might have been less effective. In pain analgesia paradigms, priors can influence outcomes bidirectionally: they may enhance pain (hyperalgesia) or diminish it (hypoalgesia), so the provided example does not fully capture the workings of priors. We assume that the likelihood, or actual pain reduction, is the same for both Patient A and Patient B, as indicated by the yellow-outlined box. From a Bayesian perspective, the posteriors for both patients differ, shown in the blue-outlined box. The graph on the right side of the figure demonstrates the Bayesian concept, assuming that all parameters follow a Gaussian distribution with equal standard deviations (noting that this is a simplification for convenience). In Patient A’s Bayesian brain model, the prior (mean VAS rating of 30) shifts the likelihood function (mean VAS rating of 50) towards a posterior mean VAS rating of 40. The same principle applies to Patient B’s model, causing a shift in the opposite direction. This diagram illustrates how varying priors can modulate individual responses to acupuncture analgesia.

We illustrated a hypothetical scenario to clarify our Bayesian brain model for acupuncture, demonstrating how varying priors can affect the outcomes of acupuncture analgesia, as depicted in Figure 2. In this figure, Patient A and Patient B have distinct prior beliefs about acupuncture (represented in the green box labeled “prior”). Patient A anticipates that acupuncture will reduce painful stimuli (depicted as red bars with numerical VAS ratings above each bar) from 100 to 30, whereas Patient B expects a reduction from 100 to 70. This difference in expectations between Patient A and Patient B may stem from diverse experiences or personal beliefs about acupuncture in clinical contexts or, in experimental settings, from different conditioning trials. The actual extent to which acupuncture treatment reduces perceived pain in both Patient A and Patient B (illustrated in the yellow box labeled “likelihood”) is identical, decreasing from 100 to 50. However, when combining the prior and likelihood into the posterior (depicted in the blue box labeled “posterior”), the prior affects the likelihood, leading to a mean shift and consequently different outcomes (or experiences) of acupuncture analgesia.

Note that the terminology used in the legend of the formula for our Bayesian brain model might differ from that in general definition. In the following section, we provide a detailed description of each element constituting our model.

### 3.1. Prior

The prior refers to our expectation of acupuncture based on prior experiences. It mainly consists of cognitive factors that influence acupuncture perception. We unconsciously weigh each component of the prior differently (see below).

$$Prior\left(t\right)=a\;*\;E+b\;*\;K+c\;*\;Ex\ldots$$

$\left(E=expectation,K=knowledge,Ex=experience,etc.;a,b,c\ldots =weight\right)$

In an experimental setting, the weight assigned to each component can be quantified using self-reported questionnaires. For example, the Massachusetts General Hospital Acupuncture Sensation Scale can be used to examine individual acupuncture sensations [40], the acupuncture expectancy scale to assess acupuncture treatment expectations [41], and the acupuncture fear scale to evaluate the fear of acupuncture [42]. However, priors include multiple factors of experience, our perception of stimuli, and the desire for favorable outcomes [43] and are not easily formed in clinical settings. In a broader perspective, priors may be influenced by the valence of induced stimuli, as attention may be directed in a biased way. For example, we are likely to incorporate good news and ignore bad news [44]. The cognitive factors influencing acupuncture sensations or therapeutic efficacies may be understood as accounting for priors.

The influence of priors on therapeutic efficacy is best explained by placebo effects and especially by open-label placebos. In the latter, although patients are truthfully informed that they are receiving a placebo, their bodies may instinctively react based on a subconscious belief that they are being administered medication [33]. The act of taking pills or undergoing needling is embedded within a medical framework in which patients seek aid, leading individuals to perceive pain relief even in a non-deceptive placebo scenario [45]. Within the framework of predictive coding, it is expected that patients will still report diminished pain sensations even if they receive placebo pills or sham acupuncture (of which they are aware). Placebo effects differ in each individual. Previous research found that there was no significant correlation between a placebo pill group, a sham acupuncture group, and a cue-conditioned group; rather, the brain activation patterns of participants in each group differed, indicating unique individual responses to a placebo [46]. Similarly, placebo needles were shown to elicit complex behavioral responses due to enhanced touch sensations, direct stimulation of the somatosensory system, and the activation of multiple brain systems, implying that factors influencing expectations are related to somatosensory aspects [47]. Therefore, since each of our Bayesian brains is wired differently (since our priors are different), our responses to placebos are likely to differ as well. This is especially the case for multi-dimensional treatment modalities such as acupuncture, in which not only somatosensory but also contextual aspects contribute significantly to inducing placebo responses. This phenomenon underscores the influence of psychological factors, especially expectations in everyday life, which are akin to narratives emphasizing broader contexts. The therapeutic encounters occurring during acupuncture are akin to rituals [48]. In other words, priors have a place in our everyday lives.

Furthermore, novel paradigms for researching cognitive influence in acupuncture, such as phantom, VGAIT, and visual acupuncture, have consistently demonstrated that, even in the absence of tactile or somatosensory stimuli, both acupuncture sensation and clinical efficacy can be induced [28,49,50]. Indeed, investigations of the predictive role of the salience network (expectation) in monitoring internal and external bodily states in the context of acupuncture have shown that expectations of acupuncture stimulation induce both a distinct somatosensory sensation (*de-qi*) and brain activation [37]. These results suggest that the non-specific effects of acupuncture influence the prior, creating predictions heavily impacted by the cognitive aspects of acupuncture.

In acupuncture perception, cognitive factors other than expectations contribute to the prior. Specifically, the doctor–patient relationship can significantly alter our prior expectation of treatment. A positive doctor–patient relationship can heighten both treatment expectancy and the placebo effect of sham acupuncture needles. Doctors’ attire, but not doctors’ facial appearance, has been shown to influence the patient–doctor relationship, suggesting that these and other cognitive factors contribute to acupuncture perception and therapeutic outcomes [12,30,51,52]. Patients with chronic pain reported lower pain intensity mediated by patient–clinician nonverbal behavioral mirroring and brain-to-brain concordance, associated with the theory of mind and social mirroring [53]. Also, pain induced by therapeutic tools (acupuncture) in the context of treatment is modulated differently in our brain, thus demonstrating the power of context in medical practice [10].

Bodily awareness and attention also influence the prior. Enhanced bodily awareness can induce *de-qi* sensations even when acupuncture is administered to a fake hand, as demonstrated in the rubber hand illusion model [17]. Similarly, enhanced bodily attention can activate the salient interoceptive–autonomic network and deactivate the default mode network, regardless of whether acupuncture stimulation was actually performed [16]. Perceived somatic sensations for acupuncture stimulation can be modulated by different cues [54]. Even imagining acupuncture (such as through phantom acupuncture, VGAIT, or visual acupuncture) can elicit acupuncture sensations and analgesia in certain parts of the body, which indicates that enhanced bodily attention can induce interoceptive sensations, along with physiological changes and acupuncture analgesia. In a Bayesian brain model, non-specific effects or cognitive factors of acupuncture perception may be defined as priors influencing our perception.

Cultural and social differences among diverse populations can also shape prior beliefs. East Asian cultures, grounded in the principles of oriental philosophy and traditional medicine, are often more open to therapies such as acupuncture and herbal medicine. In contrast, Western cultures, which are deeply rooted in contemporary anatomical and physiological understandings of the human body, may be less familiar with the concepts of oriental medicine. Consequently, treatment expectations can vary due to cultural diversity.

Overall, non-specific effects or cognitive factors influencing the perception of acupuncture, along with varied prior beliefs stemming from different cultural backgrounds, can be considered as priors that affect our perception within our Bayesian brain model.

### 3.2. Sensory Input

During the actual acupuncture experience, sensory information enters the equation, as bottom-up sensory signals gathered from the lower levels of our perception system (e.g., receptors) are activated. Although acupuncture itself inhibits pain signal transmission in peripheral and central systems—by activating different patterns of afferent fibers and opioid receptors, thereby eliciting analgesic effects [55,56]—our Bayesian brain model focuses on how sensory information is received and processed as a form of cognition. Sensory input can be equated with the somatosensory components of acupuncture and categorized as exteroception and interoception.

Exteroception encompasses all sensations that result from external stimuli, including vision, touch, and pain. During acupuncture, sensations related to needling include pricking sensations from the initial needle insertion and deep aching or dull sensations from manipulation of the needle (often referred to as “*de-qi*”). Acupuncture without tactile stimulation (visual acupuncture) should be considered as well since eliciting certain acupuncture-related sensations can result in treatment efficacy [26,28,29]. Studies have shown that exteroception can be experimentally influenced by priors through conditional cues related to pain relief, affective touch, and emotion [57,58,59,60].

Interoception refers to the perception and integration of autonomic, hormonal, visceral, and immunological signals, i.e., sensations from within the body [61]. Our perception (both conscious and unconscious) of the inner state of our body falls into this category, and in the context of acupuncture, it includes bodily ownership, enhanced local blood flow, and the distinct spatial patterns of needling sensation [62,63,64]. The role of touch can be merged in the interoceptive aspect of acupuncture as well since affective-social aspects arising from how we perceive touch are related to physiological changes in our body [65]. However, in our focus on the Bayesian brain model, we are mainly interested in how priors influence individual perceptions of acupuncture and thus do not distinguish between the various kinds of acupuncture sensations.

### 3.3. Cognitive Appraisal

Cognitive appraisal is defined as the process by which an individual perceives a stimulus and then appraises its emotional significance, which in turn triggers affective, physiological, and behavioral responses [66]. During acupuncture treatment, we assume that our brain assigns a certain valence to the stimulus. Simply put, cognitive appraisal in our Bayesian model is the process of how we perceive acupuncture as sensory information and assign affective valence.

Considering valence (or, in a broader term, emotion) is important because it tends to influence how we perceive and predict stimuli [67]. Feelings like anxiety are known to be associated with nocebo effects [68], which can diminish therapeutic outcomes, whereas feelings of reward often have the opposite effect [69]. Future research should focus on elucidating the neural basis of individual differences in responsiveness to acupuncture. It should also explore how cognitive factors such as treatment expectations, emotional valence, bodily attention, and the doctor–patient relationship influence acupuncture perception within the Bayesian framework. The valence of expectations may act as a powerful cognitive factor that modulates current experiences, as seen in pain perception [70]. In pain processing, negative expectations evoke anxiety, while positive expectations elicit rewarding responses, which demonstrates that the valence of expectations influences posterior outcomes [71]. Furthermore, emotional appraisal may either down- or upregulate early sensory processing based on the emotional valence of expectation [72]. Thus, in the Bayesian integration framework of pain perception, emotion can be expected to play a significant role in integrating expectations with sensory input. Also, emotions are known to bias behavioral and neural responses related to prediction errors [73].

Similarly, in acupuncture perception, emotion and affective valence influence how we process acupuncture sensations. Perceiving acupuncture as a reward activates reward circuitries in the brain [10]. Moreover, the perception of acupuncture elicits different psychophysiological responses to identical acupuncture stimulation depending on whether the context is positive or negative [38]. These findings suggest that, similar to pain perception, emotion plays a major role in acupuncture perception.

Introducing affective valence to appraise sensory inputs (cognitive appraisal) aligns likelihood with prior; in predictive coding, prediction errors, i.e., the difference between what we perceive and what we predict, must be calculated. Since sensory input alone cannot integrate with the prior, assigning a valence to sensory input successfully depicts how sensory input (with its valence) interacts with the prior. With this approach, the raw sensory data incorporate affective valence, that is, whether the acupuncture sensation is perceived as effective or *positive* (as a reward or treatment) vs. ineffective or *negative* (as a nociceptive stimulus). Thus, cognitive appraisal transforms low-level sensory data into high-level sensory data by encompassing valence-related aspects of acupuncture perception [74].

### 3.4. Prediction Error

The heart of the Bayesian brain model lies in how the prediction error is minimized. In the context of acupuncture, a predictive error may stem from disparities between the expected and actual sensations elicited by acupuncture stimulation as well as by how efficient we perceive acupuncture to be compared to how we have predicted it to be. The prediction error serves as a learning signal in the hierarchical generative model of predictive coding and is distributed through multiple layers, updating the brain’s internal model and influencing future responses (or priors) [75]. Usually, prediction errors are accompanied by learning rates (in Bayesian terms, *gain*), which assign a specific weight to a certain prediction error, thus affecting how the adjustment of the prediction influences the internal model predicted by the Bayesian brain [76]. Although learning rates cannot be specified in the human brain as concisely as in areas of machine learning, they serve as a broad concept of how prediction errors may influence updates in the hierarchical generative model.

## 4. Discussion

To date, existing theories on the mechanisms of acupuncture have primarily used neuroimaging to explore and interpret the various physiological responses to acupuncture, aiming to enhance our understanding of how it works [77]. Our Bayesian brain model advances this field by incorporating predictive coding theory and the Bayesian brain framework to integrate cognitive factors with our prior knowledge of acupuncture. This approach allows us to gain a deeper understanding of how acupuncture affects both the body and the brain. By employing psychophysical and psychophysiological measures within the context of acupuncture and applying these parameters within a Bayesian framework, we offer new insights into how acupuncture may function as a dynamic interplay of somatosensory input and cognitive modulation.

Our novel paradigm for acupuncture research applies predictive coding theory and the Bayesian brain model to obtain an understanding of how acupuncture works in our body and our brain. In the area of acupuncture research, progress has been made in understanding how cognitive factors influence our perception of acupuncture. Although the cognitive components of acupuncture, namely, the power of mind, rarely lead to cure on their own, they can aid in enhancing treatment effects. In clinical settings, the healing process is thought to reflect the combined power of the body, the mind, and medicine. The weight of each one differs depending on the treatment modality. In acupuncture, the power of the mind is exceptional, as our mind (our Bayesian brain) plays an essential role in integrating perceived bottom-up sensory information with top-down predictions using predictive coding [78].

Advanced brain imaging technologies have facilitated studies of the neural mechanisms behind predictive coding by illuminating specific brain activation patterns and related functional connectivity. The prediction network in our brain is thought mainly to serve domain-general predictions and is related to motor control, implicit learning, task-driven attention and execution, and social cognition [79,80]. Future research should, therefore, include the use of brain imaging techniques to elucidate the mechanisms of predictive coding in acupuncture perception. Our Bayesian model of acupuncture perception can provide a unique perspective with which to evaluate and quantify the influences of top-down cognitive components of acupuncture. The model is primarily designed to evaluate the perceived treatment efficacy of acupuncture; that is, it includes subjective areas describing how we process acupuncture-related sensations and the perceived clinical efficacy of acupuncture. Thus, caution should be taken in interpreting our Bayesian brain model for acupuncture perception, as it mainly focuses on the perceived efficacy of acupuncture, regardless of the actual, objective clinical efficacy. Differences in subjective and objective outcomes in placebo analgesia have been reported in various studies, such that subjective efficacy may not directly translate into observed objective outcomes [23]. Nonetheless, our Bayesian brain model is a perception-based approach, and its significance in acupuncture research mainly lies in understanding the mechanism and interaction of cognitive factors in acupuncture. The discrepancy and interplay between subjective and objective outcomes following acupuncture treatment is a topic for future research.

Acupuncture is much more than needling, as it consists of diverse factors that influence both the practitioner and the patient. Acupuncture research is increasingly focused on the contributions of those factors, including doctor–patient relationships, placebo effects, expectation, bodily awareness, *de-qi*, distinct spatial patterns, and others. Shedding light on the elements beyond needling is crucial because acupuncture is not just a treatment but, rather, a sophisticated healing process whose underlying mechanisms are distinct from those of other treatment modalities. Acupuncture predates modern medicine and is embedded in unique beliefs and rituals, passed on from generation to generation. The fact that placebo acupuncture differs from other placebo modalities and seems to be more strongly influenced by cognitive factors stems from the fundamental characteristic of acupuncture as a complex healing process in which the effects of needling are supported by a belief in regaining wellness and health [81]. Delving into this realm would provide a clearer view of our perception of acupuncture as an integrated high-level cognitive process. The resulting insights can be applied to augment the perceived clinical efficacy of acupuncture treatment.

The role of genetic differences or intrinsic factors in individual sensitivity to acupuncture has been explored [82]; however, in the cognitive context, individual responsiveness to acupuncture perception and thus therapeutic efficacy has not yet been thoroughly researched, despite its importance in practitioners’ attempts to evaluate their patient’s response to acupuncture. Integrating the Bayesian framework into cognitive aspects of acupuncture may explain why individuals respond to acupuncture differently. By applying predictive coding theory, we postulated that individuals differ in their priors, resulting in different internal models of acupuncture perception and, thus, ultimately in discrepant predictions that integrate with sensory information. Thus, even if practitioners administer treatments with protocoled acupoints, needling depth, and dosage, different prediction errors may arise that influence an individual’s perception of acupuncture, resulting in an individual’s responsiveness to acupuncture treatment.

Understanding individual responsiveness in clinical settings is crucial for tailoring optimal treatments for patients. Our Bayesian brain model emphasizes that individuals process information differently based on their unique priors. In practice, by understanding each person’s priors—shaped by their perceptions of acupuncture—clinicians can better anticipate how patients might react to treatment. For instance, positive cognitive factors, such as expectations of relief or past experiences confirming efficacy, can enhance an individual’s perceived effectiveness of treatment beyond the intended therapeutic effect. Conversely, negative cognitive factors, such as unpleasant interactions with a doctor or experiencing side effects, may diminish the perceived efficacy of treatment, even below the intended therapeutic effect. This concept shares similarities with the placebo or nocebo effects but is distinct in that acupuncture acts as an active component of treatment rather than an inert control.

Moreover, this approach can help mitigate adverse events by providing a more comprehensive understanding of the patient. For example, in acupuncture, some patients who have anxiety about being pricked by a needle (trypanophobia) may experience extreme faintness or dizziness after needling, akin to symptoms of vasovagal syncope. Recognizing these characteristics—or priors, in Bayesian terms—can assist clinicians in preventing these unwanted outcomes. With recent advances in neuroimaging techniques, precise measurements of priors and other parameters, validated through psychophysical and psychophysiological data, significantly enhance the predictive capability of our model. This enables it to serve as a predictor of acupuncture perception and efficacy in clinical settings. Overall, our Bayesian brain model empowers patients to experience the full efficacy of acupuncture while aiding clinicians in delivering treatment in the most effective and comfortable manner possible.

Our article has several limitations. Foremost, our Bayesian brain model is based on theoretical interpretations of the interaction between the brain, body, and acupuncture. Without concrete experimental evidence and clinical evaluation of our model’s functionality, implementing this framework in practice may be extremely challenging. The practicality of our model depends on our understanding of parameters such as priors, cognitive appraisal, and gain. Therefore, future research utilizing up-to-date neuroimaging methodologies should aim to capture psychophysical and psychophysiological data and clarify specific parameters of our model. Additionally, our model has been simplified to facilitate understanding of the Bayesian framework’s application in acupuncture. In reality, a much more complex interplay between cognitive and somatosensory components shapes our perception. Thus, a deeper understanding of the neuropsychological basis of acupuncture may be necessary to fully comprehend how Bayesian theory can be integrated into the context of acupuncture.

In conclusion, predictive coding and the Bayesian framework offer a novel approach to studying the numerous factors that contribute to acupuncture perception, with a focus on why individual responsiveness to acupuncture may differ. Future research should be aimed at elucidating the neural basis of individual differences in responsiveness to acupuncture and how cognitive factors, such as treatment expectations, emotional valence, bodily attention, and doctor–patient relationship, influence acupuncture perception in the context of the Bayesian framework.
